# Immunosuppression Adversely Affects TST but Not IGRAs in Patients with Psoriasis or Inflammatory Musculoskeletal Diseases

**DOI:** 10.1155/2012/381929

**Published:** 2012-05-16

**Authors:** Esko Tavast, Tamara Tuuminen, Sari H. Pakkanen, Mari Eriksson, Anu Kantele, Asko Järvinen, Liana Pusa, Tarja Mälkönen, Ilkka Seppälä, Heikki Repo, Marjatta Lerisalo-Repo

**Affiliations:** ^1^Department of Bacteriology and Immunology, Haartman Institute, University of Helsinki, P.O. Box 21, 00014 Helsinki, Finland; ^2^Eastern Finland Laboratory Centre Joint Authority Enterprise (ISLAB), Mikkeli District Laboratory, Porrassalmenkatu 35-37, FI-50100 Mikkeli, Finland; ^3^Division of Infectious Diseases, Department of Medicine, Helsinki University Central Hospital, P.O. Box 348, FI-00029 HUS, Helsinki, Finland; ^4^Institute of Clinical Medicine, University of Helsinki, P.O. Box 20, 00014 Helsingin Yliopisto, Finland; ^5^Department of Internal Diseases, Länsi-Uusimaa Hospital, P.O. Box 1020, Itäinen rantakatu 9, 10601 Tammisaari, Finland; ^6^Department of Dermatology and Allergology, Helsinki University Central Hospital, P.O. Box 160, HUS 00029, Helsinki, Finland; ^7^Division of Clinical Microbiology, Helsinki University Central Hospital, HUSLAB, P.O. Box 400, HUS 00029, Helsinki, Finland; ^8^Division of Rheumatology, Department of Medicine, Helsinki University Central Hospital, P.O. Box 440, HUS 00029, Helsinki, Finland

## Abstract

The performance of the interferon gamma release assays (IGRAs) and tuberculin skin test (TST) was reviewed retrospectively in patients with psoriasis, inflammatory musculoskeletal diseases, or miscellaneous inflammatory conditions. The study was carried out over a 22-month period using 109 records of patients with psoriasis (*n* = 21), musculoskeletal disease (*n* = 74), or other inflammatory conditions (*n* = 14). Forty-four (48%) of 109 patients were on immunosuppressive therapy and 38/109 (35%) on systemic glucocorticoid therapy. The agreement between the IGRAs was substantial (*κ* = 0.71) whilst that between the IGRAs and TST was low (*κ* = 0.32). Logistic regression models revealed that IGRAs associated with risk factors for latent tuberculosis infection better than TST. TST was influenced by age, BCG vaccination, sex, and glucocorticoid therapy. We found that IGRAs performed equally well with low level of indeterminate results (1-2%). IGRAs were superior to TST because the latter was influenced by BCG-vaccination status and immunosuppressive therapy.

## 1. Introduction

Patients with latent tuberculosis infection (LTBI) have an increased risk for activation of tuberculosis (TB) if they are treated with cytostatic drugs or systemic corticosteroids [1]. The risk of LTBI reactivation is further increased by the treatment with tumour-necrosis-factor-*α*- (TNF*α*-) blocking agents [2], which have been proven to be effective therapy in autoimmune conditions such as rheumatoid arthritis (RA), psoriasis and psoriasis arthritis (PsA), and ankylosing spondylitis. In RA patients the treatment with TNF*α*-blocking agents causes a 2–4-fold increase in the risk of TB [3]. In countries with a low incidence/prevalence of TB, this occurrence of TB in RA is considered to derive mainly from reactivation of LTBI. To prevent the reactivation, national guidelines have been expressed for screening and treatment of LTBI prior to starting anti-TNF*α* therapy [4–6]. 

Two commercial interferon-*γ* (IFN*γ*) release assays (IGRAs), namely, T-SPOT.*TB* (Oxford Immunotec, Oxford, UK) and QuantiFERON-TB Gold In-Tube (Cellestis Limited, Carnegie, Victoria, Australia), have been developed. Both utilize the ability of sensitized T lymphocytes to release IFN*γ* upon stimulation with synthetic peptides specific to *Mycobacterium tuberculosis. *These methods show superior diagnostic accuracy in comparison to the tuberculin skin test (TST) [7].

The present retrospective study aimed to investigate associations between the risk factors for LTBI, vaccination status, or prior immune suppression and the results of the two commercially available IGRAs, which were appropriately modified, and TST in patients with psoriasis and inflammatory musculoskeletal diseases.

## 2. Materials and Methods

### 2.1. Patients

We analyzed the results of the 109 patients treated at the Helsinki University Central Hospital between February 2007 and December 2008 who were screened for LTBI with IGRAs and with TST. All the IGRAs had been carried out at HUSLAB, Helsinki University Central Hospital, Helsinki, Finland. We reviewed the medical records and collected data on chest X-ray examinations, history of exposure to *Mycobacterium tuberculosis*, ongoing use of glucocorticoids, immunosuppressive drugs, and/or biologicals. We divided patients into three categories according to their diagnoses (Table 1). Group 1 comprised 21 (19.2%) patients with psoriasis and psoriasis arthritis. Group 2 comprised 74 (67.9%) patients with inflammatory musculoskeletal diseases. Group 3 consisted of 14 (12.8%) patients with miscellaneous inflammatory conditions.

### 2.2. Ethics Statement

The study protocol was approved by the Ethics Committee of Helsinki and Uusimaa Hospital District Area.

### 2.3. Definitions

#### 2.3.1. Partially Treated TB

Patients with a history of tuberculosis whose anti-tuberculosis therapy had not included a three-drug regimen were considered to have had a partial treatment.

#### 2.3.2. Vaccination Status

Newborns were systematically vaccinated in maternity hospitals in Finland between 1950 and 2006. Accordingly, patients born in 1950 or later were considered as vaccinated.

#### 2.3.3. Chest X-Ray Suggestive of TB

Apical scar, primary complex, fibrosing pleural changes with/without calcifications, and fibrotic parenchymal foci were the signs indicative for LTBI.

### 2.4. IGRAs

IGRAs were performed according to the standard operating procedures (SOPs) adopted for both methods in our laboratory. For each test we developed an internal quality control sample, that is, the preparation resembling the actual clinical sample that was divided into aliquots and assayed regularly.

The commercial T-SPOT.*TB* was modified and validated as earlier described [8]. Briefly, the modifications include: the following (i) results are expressed as a number of reactive spots/10^6^ lymphocytes, and the lymphocyte count from isolated PBMC preparation is calculated with an automated hematologic analyzer (Advia 60, Bayer, Germany) for cell quantification and purity assessment; (ii) an additional positive control, that is purified protein derivative (PPD) (Statens Serum Institut, Copenhagen, Denmark) that was used with each sample; (iii) the analysis is performed in duplicates.

The B-TbIFNg is a modified version of the QuantiFERON-TB Gold In-Tube [8]. The major modification was the substitution of the original enzyme immunoassay (EIA) for IFN-*γ* (Cellestis Limited, Carnegie, Victoria, Australia) by that of PeliKine Compact human EIA (Sanquin, Amsterdam, The Netherlands). The latter gave a steeper calibration curve and ensured more accurate result interpretation in the cutoff zone [8].

#### 2.4.1. The Cut-Offs

Based on our imprecision study and a pilot evaluation [8, 9], we used the following cut-offs for our routine: for the B-TbIFNg 0.35–0.5 IU/mL and for the Ly-TbSpot 25–50 reactive cells/10^6^ lymphocytes. However, to make the results comparable with other studies, the calculations were based on the cut-offs defined by the manufacturer, that is 0.35 IU/mL and 25 reactive cells/10^6^ lymphocytes, equal to 6 spots/well.

### 2.5. TST

TST was performed by properly trained operators with 2 TU of purified protein derivative (PPD, RT23, Statens Serum Institut, Copenhagen, Denmark) according to the Mantoux technique on the surface of the forearm. The result was recorded after 48–72 h, and induration ≥10 mm was used as a positive result.

#### 2.5.1. Statistical Analysis of the Data

In the absence of the “gold standard,” we evaluated the performance of Ly-TbSpot, B-TbIFNg, and TST and assessed the results according to the presence of any risk factor for LTBI: history of active tuberculosis, birth in an endemic country, or a history of a close TB contact. Chest X-ray finding suggestive of a history of TB was primarily considered as a risk factor but was omitted from the index because (i) it was associated with odds less than 1 for positive test results; (ii) when the chest X-ray findings indicated LTBI, IGRA and TST were often not requested by physicians.

The odds ratios for positive test results were calculated with univariate logistic regression. We evaluated the results in association with an attribution to a group that is, each result was compared to all other results and to potential confounding factors such as age, sex, BCG-vaccination status and medications. Covariates with significance of *P* < 0.1 in the univariate analyses and independent from each other were included.

Cohen's kappa statistic was used to assess the agreement between the tests. We used logistic regression for the analysis of factors contributing to concordance and discordance between tests pairwise. All *P*-values were two sided.

## 3. Results

### 3.1. Characteristics of Patients

Table 1 summarizes the characteristics of the study cohort. The majority of the participants were Finnishborn, 12 (11%) had a history of TB contacts, and 12 (11%) had a history of TB. Eight of 99 (8%) patients had radiological evidence for LTBI, and less than a half of the participants had received BCG vaccination (*n* = 46 (42%)). Six (6%) patients were on anti-TNF*α* blocking therapy. Ly-TbSpot test was performed in 98, B-TbIFNg in 71, and TST in 80 patients, respectively (Table 2). 49 patients had all three tests performed.

### 3.2. The IGRAs

The risk factor analysis revealed that those being born in endemic country or having a history of contact or previous TB had higher odds for positive Ly-TbSpot (OR 2.9; *P* < 0.05, Table 3) and B-TbIFNg (OR 5.0; *P* < 0.01, Table 4). Patient group or medication did not have significant effects on IGRA results (Tables 3 and 4). Females had lower odds for positive Ly-TbSpot (OR 0.4, *P* < 0.05) than men, also in multivariate analysis.

### 3.3. TST

Age correlated negatively with a positive TST reaction, OR 0.95 (*P* < 0.01), that is patients with negative TST were older (mean 65.9 years) than those with positive TST (52.4 years). Also BCG vaccination correlated moderately with TST (10 mm, *P* < 0.1). Females had lower odds for positive TST, OR 0.2 (*P* < 0.01), than men. Group 3 had lower odds for positive test results compared with the other groups, although the number of TST performed was lower in this group (Table 5).

We found an association between TST positivity and birth in TB-endemic country, sex, and inclusion in group 3. As age and BCG vaccination status were not independent, they were included as covariates separately, both showing significant association with test results. Ongoing glucocorticoid therapy had an effect on TST, OR 0.3 (*P* < 0.05), but this effect was not confirmed in multivariate analysis (data not shown).

### 3.4. Agreement between the Tests

In the pairwise analysis (Table 6), we found no significant factors explaining the disagreement between the IGRA results. The LTBI risk factors were associated with concomitant positivity in both IGRA tests.

BCG vaccination was associated with the TST +ve/IGRA −ve discrepancy. Age had a bidirectional influence on the test results. Younger persons tended to be in the TST +ve/Ly-TbSpot−ve discrepancy group, whilst advanced age was associated with TST −ve/Ly-TbSpot +ve discrepancy. Young age was associated both with TST +ve/B-TbIFNg −ve discrepancy and concomitant TST/B-TbIFNg positivity. Male gender and corticosteroid medication were associated with TST positivity.

The limitation of this analysis is the low number of Ly-TbSpot −ve/B-TbIFNg +ve and TST −ve/B-TbIFNg +ve discrepancy groups.

The IGRAs produced highly concordant results (*κ* = 0.71) (Table 6). On the contrary, TST showed poor concordance with the IGRAs (*κ* = 0.24–0.32). When the concordance of the tests was assessed for each group separately, the conclusion drawn for the whole cohort held true also for each group (data not shown).

### 3.5. Indeterminate Results

The rates of indeterminate results were 2/98 (2%) and 1/71 (1.4%) for Ly-TbSpot and for B-TbIFNg, respectively. As to the former, both patients had lymphopenia. In one of them, although the B-TbIFNg test was negative showing an acceptable PHA reactivity, we doubted its validity because this method provides neither visual assessment nor lymphocyte normalisation, as for example, Ly-TbSpot. In one patient, the B-TbIFNg was indeterminate due to nonsufficient PHA reactivity but the same sample tested in Ly-TbSpot was acceptable because the reactivities both to PHA and PPD were sufficient.

## 4. Discussion

Here, we present findings that are not consistent with some previous studies [10, 11]. We did not observe a severe impairment with ongoing glucocorticoid or immunosuppressive antirheumatic therapy on the *ex vivo* function of lymphocytes in IGRA tests. Indeed, in our practice the rates of indeterminate results were very low and were mainly attributed to lymphopenia. In our laboratory, EIA-based methods performed equally well as the ELISPOT method, although EIA-method measures only the bulk of excreted interferon gamma and does not allow visual assessment of the quality. The good performance may be partly attributed to the EIA reagents from another manufacturer providing a steeper calibration curve, which makes the reading and interpretation more reproducible at the cut-off zone [8].

 There are several studies where the IGRAs have been compared in rheumatic and psoriatic patients ([10–16]. As reported [10–17] and reviewed [7, 18, 19]), the concordance between IGRAs and TST in adults was poor. In accordance with recent studies [10, 16], our results show good correlation between the IGRAs. Similarly to Switzerland [12], almost 100% of the Finnish population born after fifties is BCG vaccinated. We have found a negative association between age and TST positivity. This goes hand in hand with the positive association between BCG vaccination and TST results. On the contrary, the IGRAs were not affected by age or BCG status. This finding corroborated results from other studies [12, 13].

Our study has some limitations: the design was retrospective and the cohort was limited in patient numbers with unequal sizes of the patient groups. Thus, the calculated statistical parameters presented here should be regarded with caution. Another limitation of the study was that the interpretation of the chest X-ray films was not always focused on finding the Ghon's complex, the only objective indication of a TB exposure. This makes the chest X-ray film inferior to the other risk factors studied. Our finding corroborates the recent findings that the chest X-ray film gives a low yield in TB screening [10, 16, 20] and is a poor predictor of LTBI, although often this component is included into the diagnostic triad for LTBI. In our observation, calcified granuloma on the chest X-ray is not a precise correlate for LTBI because it may not be sensitive enough to reveal the lung tissue abnormalities in question.

Among all risk factors the strongest for a positive IGRA outcome appeared to be a partially treated TB (OR 7.2 and 4.6 for EIA-based and ELISPOT-based techniques, resp.). Of note, this risk factor did not emerge in the TST analysis (OR 1.2: 95 CI% 0.2–12). Furthermore, birthplace outside Finland (where the burden with environmental mycobacteria is often high) emerged as a trend for a positive TST result whereas for the IGRAs this factor was less strongly associated. It is of note that our participants were heterogeneous in relation to the factor of birthplace: only 5/12 persons were born in highly endemic countries as defined by WHO [21]. Of the rest of the subjects, 7/12 were born in the countries with a moderate and highly interregional variable TB incidence, as in Estonia.

The correlation between partial TB treatment and a positive IGRA test is expected. Tapaninen et al. have observed that even complete three-drug antituberculosis regimen given decades ago may not lead to the complete attrition of earlier sensitized lymphocytes that may recognize cognate antigens upon new *ex vivo *stimulation [22]. For that reason IGRAs should not be recommended as a follow-up method to monitor treatment efficacy.

The results of the present study encourage us to recommend EIA-based techniques throughout Finland and to other areas, where the sample transportation is challenged both by long distances and changes in temperature in the north temperate zone. This method can be applied to LTBI diagnostics in the majority of cases in whom systemic biological drugs are indicated. IGRA-based methods are of no doubt superior to TST because in contrast to TST, IGRAs provide negative result that can be distinguished from a false negative result due to immunological anergy, a feature of extreme importance when testing immunosuppressed patients. We are not in favour of simultaneous TST and IGRA testing for the screening of LTBI in patients with autoimmune conditions as suggested by Bartalesi et al. [23]. In their study only 4% of subjects were BCG vaccinated which may lead to better agreement between the methods. Moreover, our analysis showed that corticosteroid therapy is negatively associated with a positive TST making this method unreliable in patients with autoimmune disorders. In case of lymphopenia, an ELISPOT-based method should be preferred because the method allows purification and concentration of lymphocytes.

## 5. Conclusions

 In the present study B-TbIFNg and Ly-TbSpot proved to be methods of choice in evaluation of LTBI.

## Figures and Tables

**Table 1:** Patient characteristics.

Variable	Total (*n* = 109)	Group* (*n* = 21)	Group 2^†^ (*n* = 74)	Group 3^‡^ (*n* = 14)
Age, mean years	57.5	51	58.7	60.9
Quartiles 25–75%	48.5–68.4	41.4–57.4	48.5–70.3	50.8–66
Sex				
Female, *n* (%)	65 (60)	8 (38)	49 (66)	8 (57)
Born in TB-endemic country^§^				
Yes, *n* (%)	12 (11)	0 (0)	9 (12)	3 (21)
Contact with TB				
Yes, *n* (%)	12 (11)	0 (0)	11 (15)	3 (21)
Previous TB-history				
No, *n* (%)	97 (89)	19 (90)	64 (86)	14 (100)
Full treatment, n(%)	4 (4)	1 (5)	3 (4)	0 (0)
Partially treated, *n* (%)	8 (7)	1 (5)	7 (9)	0 (0)
Positive chest X-ray^||^				
Yes, *n* (%)	8 (7)	0 (0)	7 (9)	1 (7)
BCG vaccination				
Yes, *n* (%)	46 (42)	16 (76)	27 (36)	3 (21)
Treatment				
Corticosteroids, *n* (%)	38 (35)	1 (5)	31 (42)	6 (43)
Cytostatics, *n* (%)	48 (44)	9 (43)	35 (47)	4 (29)
Biological, *n* (%)	6 (6)	4 (19)	2 (3)	0 (0)

*Psoriasis.

^†^Inflammatory musculoskeletal diseases: rheumatoid arthritis 38, ankylosing spondylitis 11, vasculitis 7, seronegative polyarthritis 6, oligoarthritis 4, myositis 3, juvenile idiopathic arthritis 2, chronic reactive arthritis 1, systemic lupus erythematosus 1, spondyloarthritis 1.

^‡^Miscellaneous inflammatory conditions: bronchial asthma 2, diabetes/renal transplantation 1, chronic alcholism 1, hypersedimentation 1, lung infection (mycobacterium avium-intracellulare) 1, Poncet's disease 1, Polycystic disease/renal transplantation 1, renal dysfunction 1, tubulointerstitial nephritis 1, pleuritis, lung fibrosis 1, lymphoma 1, universal arteriosclerosis 1, spinal pseudotumor 1.

^§^Cambodia, Chile, Cuba, Estonia (*n* = 2), Morocco (*n* = 2), Poland, Somalia (*n* = 2), Turkey, Ukraine.

^||^Available in 99 patients.

**Table 2:** Number of tests performed to the study population.

		Group 1 *n* = 21	Group 2 *n* = 74	Group 3 *n* = 14
Ly-TbSpot, *n* (%)	Not done	5 (23.8)*	5 (6.8)	1 (7.1)
	Neg	12 (57.1)	46 (62.2)	9 (64.3)
	Pos	4 (19.0)	22 (29.7)	3 (21.4)
	Indeterminate	0 (0.0)	1 (1.4)	1 (7.1)
B-TbIFN*γ*, *n* (%)	Not done	2 (9.5)^†^	28 (37.8)	7 (50.0)*
	Neg	17 (81.0)	34 (45.9)	5 (35.7)
	Pos	2 (9.5)	11 (14.9)	2 (14.3)
	Indeterminate	0 (0.0)	1 (1.4)	0 (0.0)
TST cut off 10 mm, *n* (%)	Not done	8 (38.1)	12 (16.2)^†^	9 (64.3)*
	Neg	4 (19.0)	28 (37.8)	4 (28.6)
	Pos	9 (42.9)	34 (45.9)	1 (7.1)

*Higher proportion of missing test results, compared with the two other patient groups, *P* < 0.05.

^†^Lower proportion of missing test results, compared with the two other patient groups, *P* < 0.05.

**Table 3:** The univariate analysis of the TB risk factors with a positive outcome of the Ly-TbSpot test.

Variable	All patients (*n* = 109)	Ly-TbSpot *n* = 98	Odds ratio	Significance
		Positive *n* = 29 (29.6)	(95% CI)	*P* <
Age, mean (test neg. mean)	57.5	56.8 (59.4)	1.0 (0.98–1.05)	0.4
Sex, female, *n* (%)	65 (59.6)	12 (41.4)	0.4 (0.1–0.9)	0.02
BCG vaccination, *n* (%)	46 (42.2)	8 (27.6)	0.4 (0.2–1.1)	0.09

Born in TB-endemic country, *n* (%)	12 (11.0)	6 (20.7)	3.3 (0.9–12.0)	0.07
Contact with TB, *n* (%)	15 (13.8)	5 (17.2)	1.4 (0.4–4.6)	0.6
Previous TB-history, *n* (%)	12 (11.0)	5 (17.2)	2.2 (0.6–7.8)	0.2
Any risk factor for LTBI, *n* (%)	33 (30.3)	14 (48.3)	2.9 (1.1–7.1)	0.03

Positive chest X-ray, *n* (%)	8 (7.3)	1 (4.0)	0.5 (0.1–4.4)	0.5

Group 1, *n* (%)	21 (19.3)	4 (25.0)	0.8 (0.2–2.6)	0.7
Group 2, *n* (%)	71 (65.1)	22 (31.9)	1.2 (0.7–2.0)	0.4
Group 3, *n* (%)	17 (15.6)	3 (23.1)	0.8 (0.5–1.2)	0.3

Corticosteroids, *n* (%)	38 (34.9)	11 (37.9)	1.0 (0.4–2.5)	1.0
Cytostatic treatment, *n* (%)	48 (44.0)	14 (48.3)	1.1 (0.5–2.6)	0.9
Biological treatment, *n* (%)	6 (5.5)	1 (3.4)	0.5 (0.1–4.1)	0.5

**Table 4:** The univariate analysis of the TB risk factors with a positive outcome of the B-TbIFNg test.

Variable	All patients *n* = 109	B-TbINF*γ* *n* = 71	Odds ratio	Significance
		Positive *n* = 15 (21.1)	(95% CI)	*P* <
Age, mean (test neg. mean)	57.5	58.7 (58.8)	1.0 (0.96–1.04)	1
Sex, female, *n* (%)	65 (59.6)	7 (46.7)	0.6 (0.2–1.8)	0.4
BCG vaccination, *n* (%)	46 (42.2)	4 (26.7)	0.4 (0.1–1.5)	0.2

Born in TB-endemic country, *n* (%)	12 (11.0)	3 (20.0)	2.6 (0.5–12.2)	0.3
Contact with TB, *n* (%)	15 (13.8)	4 (26.7)	3.0 (0.7–12.6)	0.2
Previous TB-history, *n* (%)	12 (11.0)	4 (26.7)	3.7 (0.9–16.1)	0.08
Any risk factor for LTBI, *n* (%)	33 (30.3)	9 (60.0)	5.0 (1.5–16.6)	0.009

Positive chest X-ray, *n*(%)	8 (7.3)	1 (7.1)	0.9 (0.1–9.0)	0.9

Group 1, *n* (%)	21 (19.3)	2 (10.5)	0.4 (0.07–1.7)	0.2
Group 2, *n* (%)	71 (65.1)	11 (24.4)	1.3 (0.7–2.5)	0.4
Group 3, *n* (%)	17 (15.6)	2 (28.6)	1.2 (0.6–2.1)	0.6

Corticosteroids, *n* (%)	38 (34.9)	3 (20.0)	0.5 (0.1–2.1)	0.4
Cytostatic treatment, *n* (%)	48 (44.0)	6 (40.0)	1.0 (0.3–3.3)	1.0
Biological treatment, *n* (%)	6 (5.5)	0 (0)	—	1.0

**Table 5:** The univariate analysis of the TB risk factors with a positive outcome for TST.

Variable	All patients *n* = 109	TST cut-off 10 mm		
		*n* = 80	OR	Significance
		Positive *n* = 44	(95% CI)	*P* <
Age, mean (test neg. mean)	57.5	53.0 (62.4)	0.95 (0.92–0.99)	0.006
Sex, female, *n* (%)	65 (59.6)	(47.7)	0.2 (0.06–0.5)	0.002
BCG vaccination, *n* (%)	46 (42.2)	23 (52.3)	2.4 (1.0–6.3)	0.06
Born in TB-endemic country, *n* (%)	12 (11.0)	8 (18.2)	7.8 (0.9–65.5)	0.06
Contact with TB, *n* (%)	15 (13.8)	5 (11.4)	0.5 (0.2–1.8)	0.6
Previous TB history, *n* ( %)	12 (11.0)	6 (13.6)	0.8 (0.2–2.7)	0.7
Any risk factor for LTBI, *n* (%)	33 (30.3)	15 (34.1)	0.9 (0.4–2.3)	0.9
Positive chest X-ray, *n* (%)	8 (7.3)	2 (4.9)	0.5 (0.1–3.1)	0.5
Group 1, *n* (%)	21 (19.3)	9 (69.2)	2.1 (0.6–7.3)	0.3
Group 2, *n* (%)	71 (65.1)	34 (54.8)	1.0 (0.6–1.7)	1.0
Group 3, *n* (%)	17 (15.6)	1 (20.0)	0.5 (0.2–1.0)	0.06
Corticosteroids, *n* (%)	38 (34.9)	9 (20.5)	0.3 (0.1–0.9)	0.03
Cytostatic treatment, *n* (%)	48 (44.0)	24 (54.5)	1.9 (0.8–4.6)	0.2
Biological treatment, *n* (%)	6 (5.5)	2 (4.5)	0.5 (0.1–3.3)	0.5

**Table 6:** The agreement between IGRAs and TST with negative/negative as the reference category.

		Negative	Positive	*n*	Concordance	Kappa (std. Error)
		Ly-TbSpot				

B-TbIFNg	Negative, *n* (%)	41 (64)	8 (13)	64	0.88	0.71 (0.09)
	Positive, *n* (%)	0 (0)	15 (23)			
			Any risk factor 3.6 (1.06–12)			

TST cut-off 10 mm	Negative, *n* (%)	28 (38)	5(7)	74	0.65	0.32 (0.10)
			Age 1.14 (1.01–1.29)			
	Positive, *n* (%)	21 (28)	20 (27)			
		Age 0.94 (0.90–0.98)				
		Woman 0.22 (0.057–0.87)	Woman 0.071 (0.017–0.30)			
		BCG 3.6 (1.11–12)				

		B-TbIFN*γ*				

TST cut off 10 mm	Negative, *n* (%)	19 (37)	3 (6)	51	0.61	0.26 (0.11)
	Positive, *n* (%)	17 (33)	12 (24)			
		Age 0.90 (0.85–0.97)	Age 0.93 (0.87–0.99)			
		Woman 0.17 (0.035–0.78)	Woman 0.13 (0.025–0.72)			
		BCG 4.7 (1.18–18)				
		Costeroids 0.15 (0.03–0.83) Corticosteroids 0.10 (0.01–0.94)				

## Abbreviations

BCG: Bacille Calmette-Guérin
TST: Tuberculin skin test
PTP: Pretest probability
PPV: Positive predictive value
NPV: Negative predictive value
CI: Confidence interval
IGRA: Interferon gamma release assay
PA: Proportions of agreement
*κ*: Cohen's unweighted kappa.

## References

[1] Winthrop KL, Chiller T (2009). Preventing and treating biologic-associated opportunistic infections. *Nature Reviews. Rheumatology*.

[2] Giles JT, Bathon JM (2004). Serious infections associated with anticytokine therapies in the rheumatic diseases. *Journal of Intensive Care Medicine*.

[3] Desai SB, Furst DE (2006). Problems encountered during anti-tumour necrosis factor therapy. *Best Practice and Research*.

[4] Furst DE, Cush J, Kaufmann S, Siegel J, Kurth R (2002). Preliminary guidelines for diagnosing and treating tuberculosis in patients with rheumatoid arthritis in immunosuppressive trials or being treated with biological agents. *Annals of the Rheumatic Diseases*.

[5] Ledingham J, Deighton C (2005). Update on the British Society for Rheumatology guidelines for prescribing TNF*α* blockers in adults with rheumatoid arthritis (update of previous guidelines of April 2001). *Rheumatology*.

[6] Solovic I, Sester M, Gomez-Reino JJ (2010). The risk of tuberculosis related to tumour necrosis factor antagonist therapies: a TBNET consensus statement. *European Respiratory Journal*.

[7] Pai M, Zwerling A, Menzies D (2008). Systematic review: t-cell-based assays for the diagnosis of latent tuberculosis infection: an update. *Annals of Internal Medicine*.

[8] Tavast E, Salo E, Seppälä I, Tuuminen T (2009). IGRA tests perform similarly to TST but cause no adverse reactions: pediatric experience in Finland. *BMC Research Notes*.

[9] Tuuminen T, Tavast E, Väisänen R, Himberg JJ, Seppälä I (2010). Assessment of imprecision in gamma interferon release assays for the detection of exposure to mycobacterium tuberculosis. *Clinical and Vaccine Immunology*.

[10] Kleinert S, Kurzai O, Elias J (2010). Comparison of two interferon-*γ* release assays and tuberculin skin test for detecting latent tuberculosis in patients with immune-mediated inflammatory diseases. *Annals of the Rheumatic Diseases*.

[11] Soborg B, Ruhwald M, Hetland ML (2009). Comparison of screening procedures for Mycobacterium tuberculosis infection among patients with inflammatory diseases. *Journal of Rheumatology*.

[12] Laffitte E, Janssens JP, Roux-Lombard P (2009). Tuberculosis screening in patients with psoriasis before antitumour necrosis factor therapy: comparison of an interferon-*γ* release assay vs. tuberculin skin test. *British Journal of Dermatology*.

[13] Matulis G, Jüni P, Villiger PM, Gadola SD (2008). Detection of latent tuberculosis in immunosuppressed patients with autoimmune diseases: performance of a Mycobacterium tuberculosis antigen-specific interferon *γ* assay. *Annals of the Rheumatic Diseases*.

[14] Inanc N, Aydin SZ, Karakurt S, Atagunduz P, Yavuz S, Direskeneli H (2009). Agreement between quantiferon-TB gold test and tuberculin skin test in the identification of latent tuberculosis infection in patients with rheumatoid arthritis and ankylosing spondylitis. *Journal of Rheumatology*.

[15] Jeong HP, Ga YS, Jin SL, Kim TH, Yoo DH (2009). Positive conversion of tuberculin skin test and performance of interferon release assay to detect hidden tuberculosis infection during anti-tumor necrosis factor agent trial. *Journal of Rheumatology*.

[16] Martin J, Walsh C, Gibbs A (2010). Comparison of interferon {gamma} release assays and conventional screening tests before tumour necrosis factor {alpha} blockade in patients with inflammatory arthritis. *Annals of the Rheumatic Diseases*.

[17] Tuuminen T, Sorva S, Liippo K (2007). Feasibility of commercial interferon-*γ*-based methods for the diagnosis of latent Mycobacterium tuberculosis infection in Finland, a country of low incidence and high bacille Calmette-Guérin vaccination coverage. *Clinical Microbiology and Infection*.

[18] Canada Communicable Disease Report (2010). Recommendations on interferon gamma release assays for the diagnosis of latent tuberculosis infection—2010 Update. *CCDR-RMTC*.

[19] Morbidity and Mortality Weekly Report (2010). Updated guidelines for using interferon gamma release assays to detect Mycobacterium tuberculosis infection—United States. http://www.cdc.gov/mmwr/pdf/rr/rr5905.pdf.

[20] Eisenberg RL, Pollock NR (2010). Low yield of chest radiography in a large tuberculosis screening program. *Radiology*.

[21] World Health Organization WHO Global tuberculosis control—epidemiology, strategy, financing. http://www.who.int/tb/publications/global_report/2009/en/.

[22] Tapaninen P, Korhonen A, Pusa L, Seppälä I, Tuuminen T (2010). Effector memory T-cells dominate immune responses in tuberculosis treatment: antigen or bacteria persistence?. *International Journal of Tuberculosis and Lung Disease*.

[23] Bartalesi F, Vicidomini S, Goletti D (2009). QuantiFERON-TB GOLD and the TST are both useful for latent tuberculosis infection screening in autoimmune diseases. *European Respiratory Journal*.

